# Monitoring of Water Content in Building Materials Using a Wireless Passive Sensor

**DOI:** 10.3390/s100504270

**Published:** 2010-04-28

**Authors:** Goran Stojanović, Milan Radovanović, Mirjana Malešev, Vlastimir Radonjanin

**Affiliations:** Faculty of Technical Sciences, University of Novi Sad, Trg Dositeja Obradovića 6, 21000 Novi Sad, Serbia; E-Mails: rmilan@uns.ac.rs (M.R.); miram@uns.ac.rs (M.M.); radonv@uns.ac.rs (V.R.)

**Keywords:** LC sensor, wireless, humidity, building materials

## Abstract

This paper describes an innovative design of a wireless, passive LC sensor and its application for monitoring of water content in building materials. The sensor was embedded in test material samples so that the internal water content of the samples could be measured with an antenna by tracking the changes in the sensor’s resonant frequency. Since the dielectric constant of water was much higher compared with that of the test samples, the presence of water in the samples increased the capacitance of the LC circuit, thus decreasing the sensor’s resonant frequency. The sensor is made up of a printed circuit board in one metal layer and water content has been determined for clay brick and autoclaved aerated concrete block, both widely used construction materials. Measurements were conducted at room temperature using a HP-4194A Impedance/Gain-Phase Analyzer instrument.

## 1. Introduction

The measurement of water content in building materials is of particular interest to professionals engaged in maintaining buildings, identifying defects and rectifying possible problems. Good quality measurements of moisture can help building professionals to take appropriate decisions about interventions and remedial work to address underlying problems [1]. Development of new methods of monitoring and measuring the moisture content of construction materials is both commercially and scientifically important. Wireless sensors allow otherwise impossible sensor applications, such as monitoring dangerous, hazardous, unwired or remote areas and locations. Application of wireless technologies also reduces maintenance complexity and costs [2]. Sensors integrated into structures, machinery, and the environment, coupled with the efficient delivery of sensed information, could provide tremendous benefits to society [3–5]. Potential benefits include: fewer catastrophic failures, conservation of natural resources, improved manufacturing productivity, improved emergency response, and enhanced homeland security [6–9]. In the construction industry there is a need to monitor a number of parameters such as the impact of vibration, humidity, material fatigue, and others, to have insight into the building state. In that way it ensures a complete rehabilitation at the time, especially for objects that are vulnerable to environmental influences [10–12].

Passive, wireless sensor which consists of inductor and interdigital capacitor (IDC) is used as a sensor for a large number of measurements. Estimation of certain parameters of the environment such as the humidity, the concentration of some gases, *etc*. can be easily calculated from changes in the capacitance of the IDC. The usual LC sensor design is constructed from the spiral inductor placed around an interdigital capacitor [13–15]. The major drawback of this solution is that if the inductive structure is realized as a square spiral inductor it is necessary to have one underpass or overpass conductor for connecting the inner terminal to the outer contact. This requires the soldering one extra wire or adding another layer of a conductor [16] and consequently complicates the fabrication process and increases the production costs of the sensor. Furthermore, if this overpass is implemented with a discrete wire, smooth and good contact between the sensor and the building material is difficult (or almost impossible) and it can be a problem during measurement.

In this paper, a completely new approach to design passive wireless sensors is presented. The whole LC sensor is made in one conductive layer and there is no need for any post-soldering process or adding extra conductive layers. This simplifies the fabrication and measurement process as well as reduces the sensor’s ultimate cost. Apart from the new sensor design, we also present the application of this wireless, passive, resonant-circuit for monitoring of water content in building materials, clay brick and autoclaved aerated concrete block, which are widely used in civil engineering.

## 2. Design of the LC sensor

The proposed wireless sensor is designed for measuring of water content in the building materials. The main features of the sensor are a simple design, low fabrication costs and it does not require any power source. The sensor was made in one metal (copper) layer providing excellent adhesion to tested building materials (a good contact between the sensor and the material being measured the water content is essential). It is also possible to insert the sensor in building materials (concrete, mortar, *etc*.) during the construction of walls or floors and after that with a handheld instrument to measure the resonant frequency and based on the measured data to monitor water content and decide if there is a need for the appropriate protection against moisture. Moreover, possible applications could be the measurement of moisture in various building materials (wood, thermal insulation, concrete, mortar) and certain structure elements of heritage buildings, indoor pools, masonry buildings, and in many other places and circumstances where there is a need to have insight into the dampness change. Data on the amount of absorbed water are important in civil engineering practice because moisture infringes insulation properties, reduces the frost resistance and accelerates the deterioration processes of building materials.

**Figure 1.**
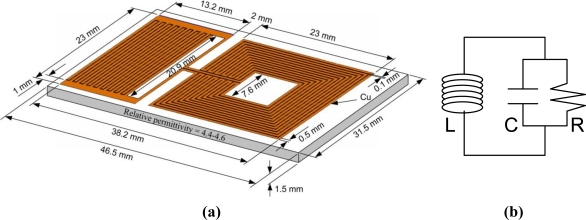
(a) The innovative design of the wireless sensor and its dimensions. (b) An equivalent electrical model of the sensor.

Figure 1(a) shows an innovative design of the LC sensor and its dimensions. The inner and outer lengths of the square, thirteen-turn spiral inductor were 7.6 and 23 mm, respectively. The interdigital capacitor had 18 sets of electrodes (fingers) of 20.9 mm (*l*) in length. The conductive lines width (*w*) of the inductor and the capacitor were 0.5 mm. The spacing (*s*) between two adjacent electrodes or capacitor’s fingers was 0.1 mm. The thickness of the copper on the circuit board was approximately 30 μm. Total mass of the sensor is 5.37 grams. The surface of the inductive part of the sensor can be coated with a thin layer of paraffin wax, which thickness is around 500 μm in order to prevent shorting the circuit when condensing water on the sensor. The sensor is modeled as an inductor *L* and capacitor *C*, connected as it is shown in Figure 1(b). The capacitive losses are represented by the resistor *R* [17].

**Figure 2.**
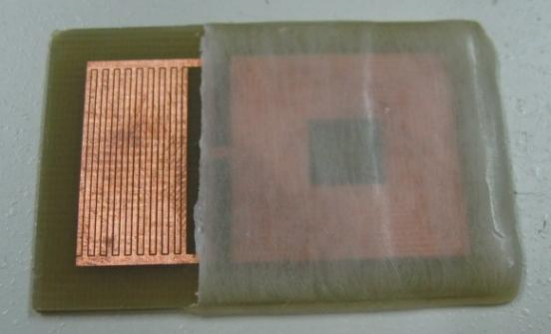
The top view of the fabricated LC sensor.

The sensor was made on a single-sided printed circuit board (PCB) with copper as a conductive material. When the capacitive part of the sensor absorbs water, its effective dielectric constant increases due to the high dielectric constant of water (about 70–80). The variation in the effective dielectric constant changes the capacitance of the IDC, resulting in the shift of the sensor’s resonant frequency and allowing tracking of water content or humidity levels. The top view of the fabricated sensor is shown in Figure 2. A thin layer of paraffin wax can be also seen, covering the inductive part of the sensor, preventing the short circuit between the inductor’s turns.

## 3. The Experimental Setup

### 3.1. Description of Experimental Setup

**Figure 3.**
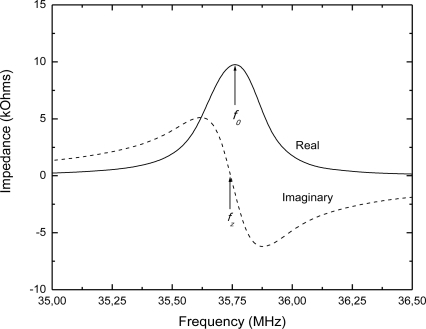
The real and imaginary part of the impedance spectrum of the designed LC sensor.

The sensor’s resonant frequency has been determined by measuring the impedance of the detection coil (placed around the sensor) with an impedance analyzer. Figure 3 shows the typical background-subtracted sensor’s impedance, where the resonant frequency *f_0_* is defined as the frequency at the maximum of the real part of the impedance (resistance), and the zero-reactance frequency *f_z_* is the frequency where the imaginary part of the impedance (reactance) goes to zero. Referring to Figure 1(b), the resonant frequency and zero-reactance frequency are related to the *R*, *L* and *C* values as given by:

(1) $$f_{0}=\frac{1}{2\pi \sqrt{\mathrm{LC}}}$$

(2) $$f_{z}=\frac{1}{2\pi }\sqrt{\frac{1}{\mathrm{LC}}-\frac{1}{R^{2}C^{2}}}$$

Measured values of the inductance of the inductive part of the LC sensor and the capacitance of the capacitive part (fabricated on separate PCB substrates) were 1.57 μH and 12.71 pF, respectively. In accordance with these values and using Equation (1), the calculated resonant frequency (without moisture) is equal to 35.64 MHz. The measured resonant frequency of the whole LC sensor had the value of 35.76 MHz (Figure 3), what is in a good agreement with the calculated value.

**Figure 4.**
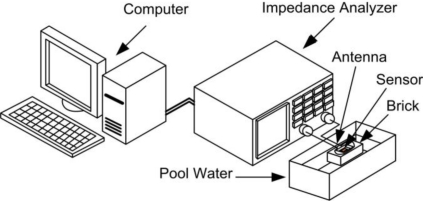
Experimental setup for testing wireless LC resonant sensor.

The experimental setup is illustrated in Figure 4. The resonant frequency of the sensor has been measured using a loop antenna that is connected to the impedance analyzer HP-4194A. Impedance analyzer HP-4194A is computer-controlled via GPIB interface. Monitoring physical parameters of materials through impedance spectroscopy has many advantages, such as: non destructive measurement, with no risk and cheap, easy to use and comparable with other monitoring alternatives.

### 3.2. Building Materials and Measuring Procedure

Measurements were made for two types of building materials, clay brick and autoclaved aerated concrete block. Clay brick is the traditional building material which is used for masonry structures, mainly for masonry walls. Phases of a clay brick manufacture are as follows: extraction of the raw material, mixing and forming processes, drying, firing, packing and distribution. The brick used in the testing has dimensions 250 × 120 × 65 mm. This product is masonry element without perforations, classified as HD brick (density 1,400 kg/m^3^) for protected masonry according to EN 771-1 [18].

Autoclaved aerated concrete is a relatively new building material which is widely used today for building of masonry walls with improved thermal properties and lower mass than traditional masonry products. Aerated concrete is made from natural raw materials–sand, lime, cement, water and aerating agent. Density of the autoclaved aerated concrete is 500 kg/m^3^ and the block that was used in the testing had dimensions of 230 × 200 × 100 mm. The intended use of autoclaved aerated concrete blocks is for protected masonry according to EN 771-4 [19]. Both chosen building materials have capillary porous structures. Additionally, autoclaved aerated concrete has cellular pores.

Some of these building materials has been set in the pool water (Figure 4). The LC sensor was positioned in the middle of the brick (for example). A loop antenna was placed around the sensor structure and the other terminals of antenna were connected to the HP-4194A Impedance/Gain-Phase Analyzer. Building brick was standing on ceramic coasters to ensure that brick would be in the water around 10 mm in height. Measurements were made in two cycles during the absorption of water from the pool, and during the drying cycle. The water absorption of the clay brick (or autoclaved aerated concrete) was done with weight measurements. Namely, the weight of the dry clay brick was first measured, with very precise scales, before putting the brick in the water pool. When the brick was set in the water pool, after certain period (around two days), the brick was in saturation (could not absorb more water). Afterwards, the measurement of the resonant frequency of the LC sensor (in the middle of the brick) and weight measurements of the building material were done. Through the ratio of the weight of wet and dry brick, the percentage of the water absorption has been calculated.

### 3.3. Operating Principles

The planar spiral inductor, together with the interdigital capacitor electrodes, forms a planar structure that can be attached to the surface or embedded in building materials for moisture measuring. Inductive and capacitive part of the sensor work together as an LC resonator whose resonant frequency is designed to change correspondingly with a change in the water content. The frequency response of the sensor is remotely detected by monitoring the impedance across the terminals of the reader antenna which actually also plays the role as a power transmitter by sending out oscillating magnetic field to the sensor by inductive link. The changes in the resonant frequency can be monitored as a result of the variation of sensor capacitance due to the changing dielectric constant of the IDC as a consequence of moisture exposure.

The basic element for detection of the water content in the presented passive sensor is an interdigital capacitor as one of the most commonly used periodic structures for sensor applications. The capacitance of an interdigital capacitor depends on the electrode gap (*s*), finger length (*l*) and width (*w*), the spatial wavelength (*λ* = 2(*w* + *s*)), metallization ratio (*η* = 2*w*/*λ*) and dielectric constant of the material between the fingers. Geometrical characteristic of the electrodes are not changed during the operation of the sensor. The sensitivity of the device is dependent on the changing properties of the dielectric layer (that means water content in our case) situated among electrodes (or fingers).

Wireless operation of the proposed LC sensor is achieved through mutual inductance coupling between the inductor on the sensor and the loop antenna. The magnetic field generated by the transmitting loop antenna induces an electromotive force across the sensor’s inductor, which generates a back-electromotive force on the antenna. Since the sensor contains LC circuit, the electromotive force on the sensor is maximal at *f_0_*, consequently the back- electromotive force in the antenna is also maximal at *f_0_* (Figure 3).

The resonant frequency of the sensor is not influenced by the distance of the wireless reading. When the reading distance is increased, the inductive coupling is reduced and consequently the magnitude of the measured impedance decreases. The reading distance was around 30 cm and approximately 7 cm diameter of loop antenna was used to stimulate the LC circuit of the sensor (the larger antenna would receive more noise from the environment). This external transmitting antenna had five turns. The impedance of the antenna is measured with high resolution using the HP-4194A instrument.

The possible sources of error in the described measurements are: corrosion of the copper conductive lines of the intedigital capacitor during the long period of exposure to the water (prevention of this effect can be achieved through covering just the surface of the capacitor “fingers” with protective lacquer), local temperature variations and to some extent the presence of metallic components near to the measurement place. We have also tried to investigate the influence of salt water on the measured results. No deposition of the salts on the sensor was observed during the drying process, but the characteristics of the building material were changed. A small hysteresis has been observed between results obtained from two cycles, during the absorption of salt water from the pool and during the drying process. The reason for this phenomenon is that salt in its solid form (during the drying cycle in the brick) does not have conductive properties, whereas solution of water and sodium chloride (in our experiment) is the electrolyte.

## 4. Results and Discussion

**Figure 5.**
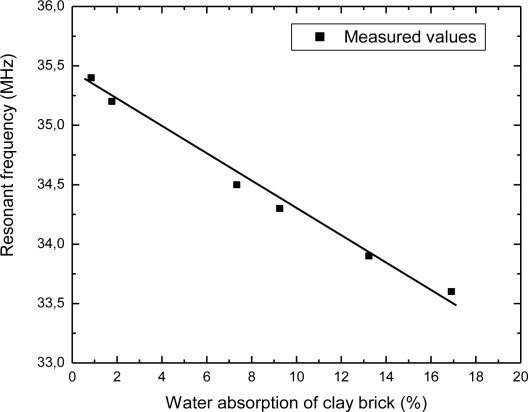
The variation of the sensor’s resonant frequency as a function of water absorption of clay brick.

**Figure 6.**
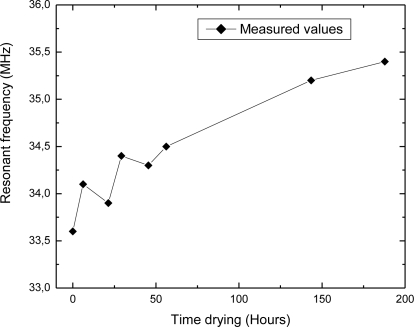
Resonant frequency as a function of time during drying brick at room temperature.

Using the experimental setup shown in Figure 4, the change in the sensor’s resonant frequency was measured as a function of time. In this section, measured results will be presented. Figure 5 depicts the dependence of the resonant frequency as a function of the percentage of water content in building bricks. From these data can be observed that the resonant frequency is changed from 35.4 MHz to 33.6 MHz, while the percentage of water absorption in the clay brick is increased from 1% to 17%. The variation of the resonant frequency was 1.8 MHz (≈ 5%), and water concentration was changed by 16%. Deviation of the measured values from the ideal linear dependence is very small (maximum error is 0.29%). Figure 6 represents dependence of the resonant frequency as a function of drying time. The time required for complete drying clay bricks at room temperature was about 190 hours. The resonant frequency of the sensor increased overtime, indicating the drying cycle of the brick. Presence of moisture increases the overall dielectric constant between the electrodes of interdigital capacitor, and *vice versa*. During the drying of the brick water started to evaporate, both dielectric constant and capacitance was decreased, and consequently the resonant frequency increased as can be seen in Figure 6.

**Figure 7.**
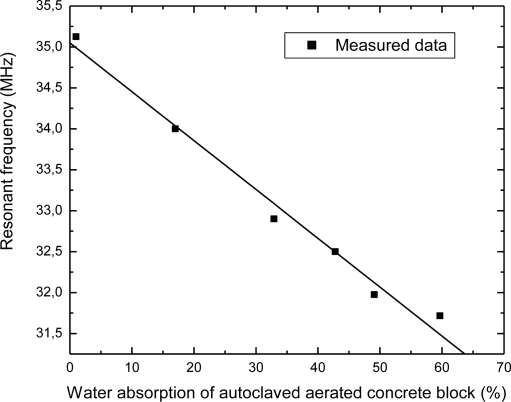
The change in the sensor’s resonant frequency as a function of water content in autoclaved aerated concrete block.

**Figure 8.**
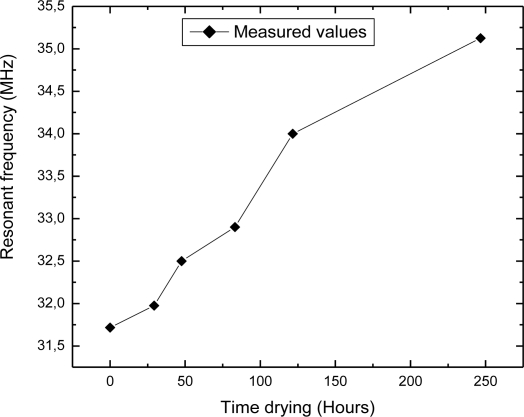
Resonant frequency as a function of time during drying autoclaved aerated concrete block at room temperature.

Dependence of the resonant frequency as a function of the water content in autoclaved aerated concrete block is presented in Figure 7. It can be seen from this figure that the resonant frequency changed from 35.1 MHz to 31.8 MHz, while the percentage of absorbed water varied in the range from 1% to 59%. The shift of the resonant frequency was 3.3 MHz (≈9.4%) when the water concentration was changed by 58%. Deviation of the measured values from the ideal linear dependence is approximately 0.68%. Dependence of the resonant frequency as a function of time during drying autoclaved aerated concrete block is depicted in Figure 8. The time required for drying autoclaved aerated concrete block at room temperature was about 250 hours. This is longer than in the case of clay brick tanks to the additional cellular pores of autoclaved aerated concrete block.

Autoclaved aerated concrete block has almost three times greater water absorption capacity than clay brick due to its more porous structure. The resonant frequency of the sensor in clay brick was lower than that in autoclaved aerated concrete block (just for illustration, for 15% of water absorption *f_0_* = 33.72 MHz for clay brick, whereas *f_0_* = 34.12 MHz for autoclaved aerated concrete block) since clay brick was denser and had a higher effective dielectric constant.

From the presented graphs it can be concluded that the proposed LC sensor can be applied for monitoring water in the walls and floors of building structures, which are located in wet areas. Continuous monitoring of moisture in building materials will enable an efficient management, rehabilitation and maintenance of buildings, churches, bridges, and other very important objects in civil engineering.

Furthermore, the described technique can be used for monitoring changes in the moisture state of building materials or relative moisture content and it is useful for many applications, for example monitoring the drying out of a building after a flood. However, some applications may also require the absolute measurement of moisture content. The presented method can be applied for this measurement, too. The calibration function between the resonant frequency and water content has been provided using stored material characteristic curves in a commercial Testo 606-2 instrument [20]. This instrument measures the material moisture of wood and building materials quickly and non-destructively and it is equipped with characteristics curves for most often used building materials such as cement screed, aerated concrete, solid brick, vertical hole brick, *etc*.

When we compare performance of the presented sensor with already reported humidity sensors, it can be concluded that our sensor has significant advantages such as simple design and cost-effective fabrication, good sensitivity and resolution, robustness and reliability. For example, in [13] the authors described a humidity sensor fabricated by sol-gel dip coating a 170 μm thick layer of TiO_2_ on an interdigital capacitor structure (a more complicated fabrication procedure than in our case). That sensor demonstrated a frequency shift of around 14 % when the humidity level was changed from 0 to 98% (this is similar to our experiment). Furthermore, a non-invasive method of measuring humidity within construction structures that is fast and low-cost was presented in [21]. The sensor consists of a printed circuit board with a planar coil on one side and a partial ground plane on the other as well as a surface mount capacitor (our LC sensor is completely planar on one sided PCB) connected in parallel with the inductor to set the resonance frequency between 7.4 MHz and 8.8 MHz. The paper [22] reported a small passive wireless humidity monitoring system that was composed of a high-sensitivity capacitive humidity sensor that forms an LC tank circuit together with a hybrid coil wound around a ferrite substrate (using ferrite increases production costs). Additionally, new sensor technologies such as nanotechnology-based sensors (multiwall carbon nanotubes show strong attraction for water moisture) can be used to measure variables that until recently were too difficult or expensive to sense and also to fabricate sensors with very small dimensions [15].

## 5. Conclusions

There is an ongoing demand for small and reliable electronic sensors capable of monitoring water content in civil engineering materials and structures since excess water may accelerate their degradation. An additional advantage is gained if the wireless sensors are passive, without an internal power supply such as battery, to avoid sensor battery life-time issues and to minimize cost. In this paper, a wireless, passive sensor for monitoring of water content for use in civil construction applications was presented. The innovative design, operating principles, experimental setup and measured parameters were described. The cost-effective sensor was made of a planar inductor-capacitor resonant circuit in one metal layer on PCB substrate. The sensor’s resonant frequency was linearly proportional to the water content in building materials. It has been demonstrated that fabricated LC wireless sensor can be successfully used for monitoring water content in materials such as clay brick and autoclaved aerated concrete block.
